# Effects of aging on foot pedal responses to visual stimuli

**DOI:** 10.1186/s40101-020-0213-2

**Published:** 2020-02-14

**Authors:** Emi Yuda, Yutaka Yoshida, Norihiro Ueda, Itaru Kaneko, Yutaka Miura, Junichiro Hayano

**Affiliations:** 1 Tohoku University Graduate School of Engineering, Aoba 6-6-05 Aramaki Aoba-ku, Sendai, 980-8759 Japan; 2 Nagoya City University Graduate School of Design and Architecture, Kita Chikusa 2-1-10 Chikusa-ku, Nagoya, 464-0083 Japan; 3 Department of Medical Education, Nagoya City University Graduate School of Medical Sciences, 1 Kawasumi Mizuho-cho Mizuho-ku, Nagoya, 467-8601 Japan; 4 Shigakkan University, 55 Nakoyama, Yokonemachi, Obu, Aichi 474-8651 Japan; 5 Department of Medical Education, Nagoya City University Graduate School of Medical Sciences, 1 Kawasumi Mizuho-cho Mizuho-ku, Nagoya, 467-8601 Japan

**Keywords:** Aging, Older driver, Driving simulator, Hesitation, Response time, Simon effect, Technoadaptability

## Abstract

**Background:**

Car accidents due to unexpected forward or backward runaway by older drivers are a serious social problem. Although the cause of these accidents is often attributed to stepping on the accelerator instead of the brake, it is difficult to induce such pedal application errors systematically with usual drive simulators. We developed a simple personal computer system that induces the pedal errors, and investigate the effects of age on the error behaviors.

**Methods:**

The system consisted of a laptop computer and a three-pedal foot mouse. It measured response time, accuracy, and flexibility of pedal operation to visual stimuli. The system displayed two open circles on the computer display, lighting one of the circles in a random order and interval. Subjects were instructed to press the foot pedal with their right foot as quickly as possible when the circle was lit; the ipsilateral pedal to the lit circle in a parallel mode and the contralateral pedal in a cross mode. When the correct pedal was pressed, the light went off immediately, but when the wrong pedal was pressed, the buzzer sounded and the light remained on until the correct pedal was pressed. During a 6-min trial, the mode was switched between parallel and cross every 2 min. During the cross mode, a cross mark appears on the display. The pedal responses were evaluated in 52 subjects divided into young (20–29 years), middle-aged (30–64 years), and older (65–84 years) groups. Additionally, the repeatability of the pedal response characteristic indicators was examined in 14 subjects who performed this test twice.

**Results:**

The mean response time was 95 ms (17%) longer in the older group than in the young group. More characteristically, however, the older group showed 2.1 times more frequent pedal errors, fell into long hesitations (response freezing > 3 s) 16 times more often, and took 1.8 times longer period to correct the wrong pedal than the young groups. The indicators of pedal response characteristics showed within-individual repeatability to the extent that can identify the age-dependent changes.

**Conclusions:**

Hesitations and extended error correction time can be associated with increased crash risk due to unexpected runaway by older drivers. The system we have developed may help to uncover and evaluate physiological characteristics related to crash risk in the elderly population.

## Introduction

With the rapid aging of society, serious car accidents caused by elderly drivers are becoming a social problem in many developed countries [1, 2]. According to a report from the Japanese National Police Agency [3], the number of fatal car accidents per 100,000 licensed population in Japan is 3.7 per year for those under 75 years old but is 5.7 for 75–79 years, 9.3 for 80–84 years, and 14.6 for those of 85 years and older. The report [3] also shows that the most common cause of traffic fatalities by older drivers is the improper operation, accounting for 31% in fatal accidents by older drivers (≥ 75 years) versus 16% by younger (< 75 years) drivers. Furthermore, among the improper operations, the pedal error of stepping on the accelerator instead of the brake accounts for 20% in the older drivers, while it accounts only for 5% in the younger drivers. Earlier studies using driving simulators have reported the ample evidence of age-related decline in driving ability and an increase in crash risk [4–11]. In driving simulators based on realistic scenarios, however, pedal operations causing a car runaway that can lead to fatal accidents are rarely triggered, making accurate quantitative assessment difficult [12]. To quantitatively characterize the pedal operation errors, methods that can induce serious pedal errors effectively and more frequently are necessary.

In this study, we developed a simple system consisting of a laptop computer and a three-pedal foot mouse to measure response time (RT), accuracy, and flexibility of pedal application to visual stimuli. The system displayed two open circles on the computer display, lighting one of the circles in a random order and interval. The subjects were instructed to press the ipsilateral or contralateral foot pedal, depending on the operation mode, with their right foot as quickly as possible when the circle was lit. Theoretically, pedal operation errors that may be related to unexpected car runaway include incorrect pedal, prolonged hesitation without proper pedaling, and delay in correcting the wrong pedal [13]. We hypothesized that these kinds of pedal errors are more frequently induced in older peoples than in younger peoples. To examine this hypothesis, we compared the characteristics of pedal responses induced by this system among young, middle-aged, and elderly subjects. In addition, we examined the reproducibility of the pedal response characteristic indicators to confirm the reliability of this method.

## Methods

### Apparatus

We developed a system called Pedal Selective Psychomotor Vigilance Test (PS-PVT). The system consisted of a laptop computer (Let’s note CF-N10, Panasonic Co., Osaka, Japan) with Microsoft Windows 7 operating system, a three-pedal foot mouse (RI-FP3BK, Route-R co., Ltd., Tokyo, Japan), and custom-made software created with Microsoft Visual Basic (Microsoft, Co., USA). The software is available from the corresponding author on a reasonable request.

**Fig. 1:**
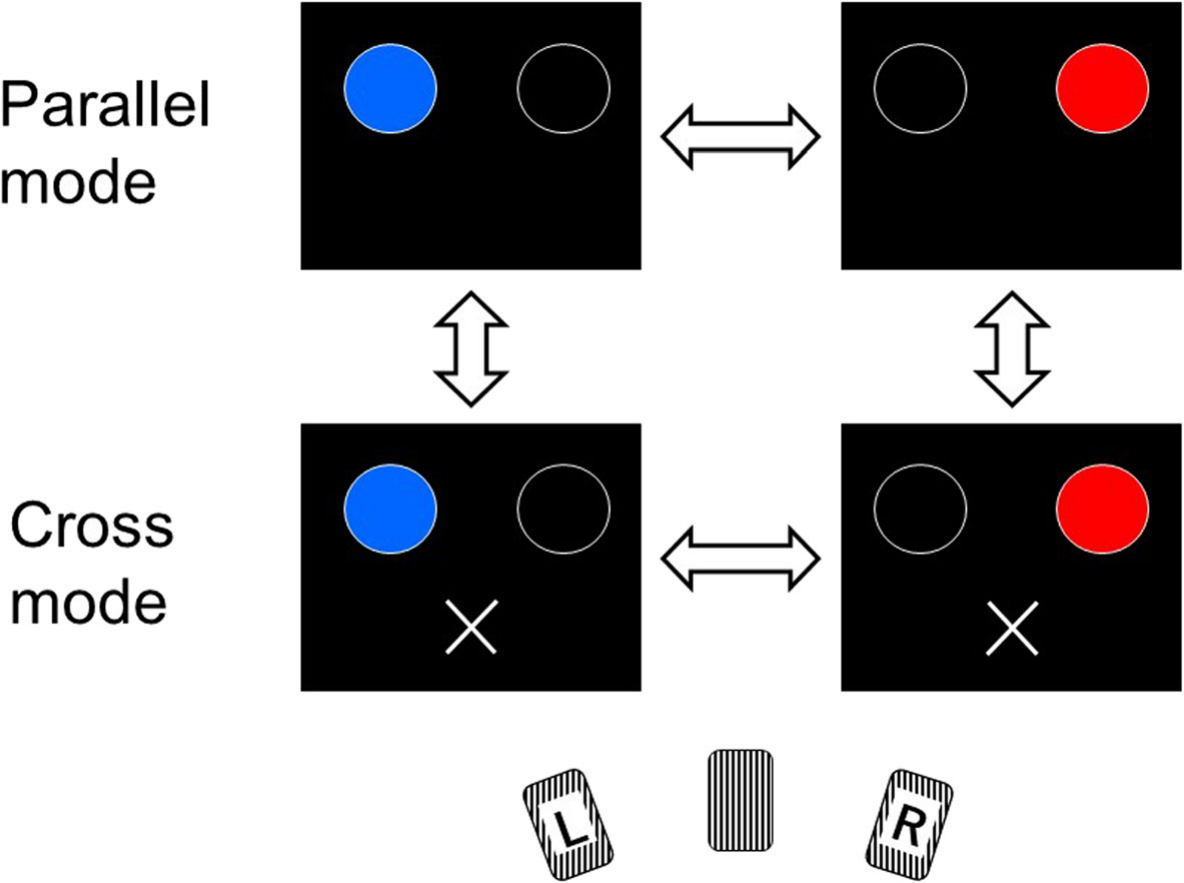
Schema of pedal selective psychomotor vigilance test (PS-PVT)

Initially, two open circles with a diameter of 5 cm were displayed horizontally on the computer display with a center-to-center distance of 15 cm (Fig. 1). Then, either one of the circles was lit in random order and interval, blue for the left circle and red for the right circle. The subjects were instructed to press a foot pedal with their right leg as prompt as possible when the circle illuminated. In parallel mode, they were asked to press the pedal ipsilateral to the lit circle, while in cross mode, they were asked to press the contralateral pedal. When the correct pedal was pressed, the light turns off immediately, but when the wrong pedal was pressed, the light did not turn off and instead, the buzzer sounded until the correct pedal was pressed. The trial lasted 6 min, during which time the mode was switched between parallel and cross every 2 min. During the cross mode, a cross mark appears on the display (lower panels of Fig. 1).

During the trial, the system recorded when and which circle was lit, when and which pedal was pressed, and the operation mode of the time. From these records, six indicators concerning the pedal response characteristics were calculated. The definition of each indicator is shown in Table 1.

**Table 1:** Indicators of pedal response characteristics evaluated by PS-PVT

Indicator	Definition
Mean response time	Average response time (RT) excluding hesitation, ms
Pedal error frequency	Relative frequency of the wrong pedal to total responses, %
Hesitation frequency	Relative frequency of hesitation (response freezing > 3 s), %
Hesitation length	Mean RT of hesitation, s
Error correction time	Time to press correct pedal after wrong pedal, ms
Cross-mode effect	The difference in RT between parallel and cross modes, ms

We estimated that the system time measurement error is at most 15 ms. Microsoft Windows 7 runs the clock with 10 to 15 ms for thread switching. The system on Visual Basic runs on a single thread, and the experiment was done while this thread had primary priority. Because we designed the software to start the measurement and timer at the same time, there was only a small error in the measurement start time. The stop timing was measured in the event handler of an input device. Assuming that all event handlings are usually done in the single process switching clock, error in measurement was expected to be at most 15 ms. It seemed enough for the experiment in this study.

### Subjects

#### Aging effect study

The PS-PVT was performed in 52 healthy subjects aged 21–84 years, including 17 women. Height was 163–178 cm in men and 148–165 cm in women. Corrected binocular visual acuity was 0.9–2.0 in men and 0.7–1.5 in women. No one has complained of mental or physical problem that hinders locomotion or other daily activities. In subjects 65 years or older, health status was confirmed by SF-36v2 [14], which showed physical, mental, and role-social component summary scores (50 ± 10 for the Japanese general population [15]) of 47 ± 7, 60 ± 7, and 52 ± 7, respectively. All subjects had a Japanese ordinary driver’s license.

#### Reproducibility study

To confirm the reliability of the pedal response characteristic indicators, PS-PVT was performed twice on the same days in the other 22 subjects aged 21–64 years including two women, and the within-individual reproducibility of the indicators was examined.

### Protocols

#### Aging effect study

The 52 subjects were divided into three groups by age as shown in Table 2. The PS-PVT was performed between 10:00 and 17:00 in a quiet air-conditioned room at 23–25 °C. Subjects sat on a chair that was adjustable to a height of 43–53 cm, facing a 70-cm high desk. The PS-PVT laptop computer was placed on the desk, and the foot pedal was placed horizontally on the floor near the feet. After receiving instructions on the PS-PVT method, subjects practiced it in parallel mode for 1 min and confirmed that he/she could do it. The height of the chair and the location of the foot pedal have been adjusted to make it easier for the subject to operate. The laptop computer was placed at an angle so that the surface of the display was perpendicular to the line of sight at a distance of 45 cm from the subject’s eye. Then, a 6-min experimental trial was performed while recording the responses. Half of the subjects in each age group started with the parallel mode and the rest of them started with the cross mode to counterbalance the effect of the operation mode order.

**Table 2:** Age groups of study subjects

Age category	*N*	Age, years	Female
Young, 20–29 years	24	21 ± 1	6
Middle, 30–64 years	11	48 ± 12	4
Older, 65–85 years	17	73 ± 6	7

#### Reproducibility study

In the same way as the aging effect, the 22 subjects performed two PS-PVT trials in the morning and afternoon at 3-h intervals. Half of the subjects started with the parallel mode, the rest of them started with the cross mode, and each subject performed in the same mode order in the morning and afternoon.

## Data and statistical analyses

The response record of each trial was processed with custom-made software to calculate the six indicators listed in Table 1.

Statistical analyses were performed with Statistical Analysis System program package (Version 9.4, SAS Institute Inc., Cary, NC, USA). The effects of age group, sex, and interaction between them were evaluated with ANOVA using the General Linear Model procedure. When the group effect was significant, multiple comparisons were performed. Data were presented in mean and the standard error of the mean. *P* < 0.05 was considered to be statistically significant and Bonferroni adjustment was used to keep the type 1 error level in multiple comparisons.

The reproducibility of indicators was evaluated by the Bland-Altman plot [16]. The 95% confidence limits (mean difference ± 1.96 SD) of differences between the two measurements were used as the limits of repeatability.

## Results

**Fig. 2:**
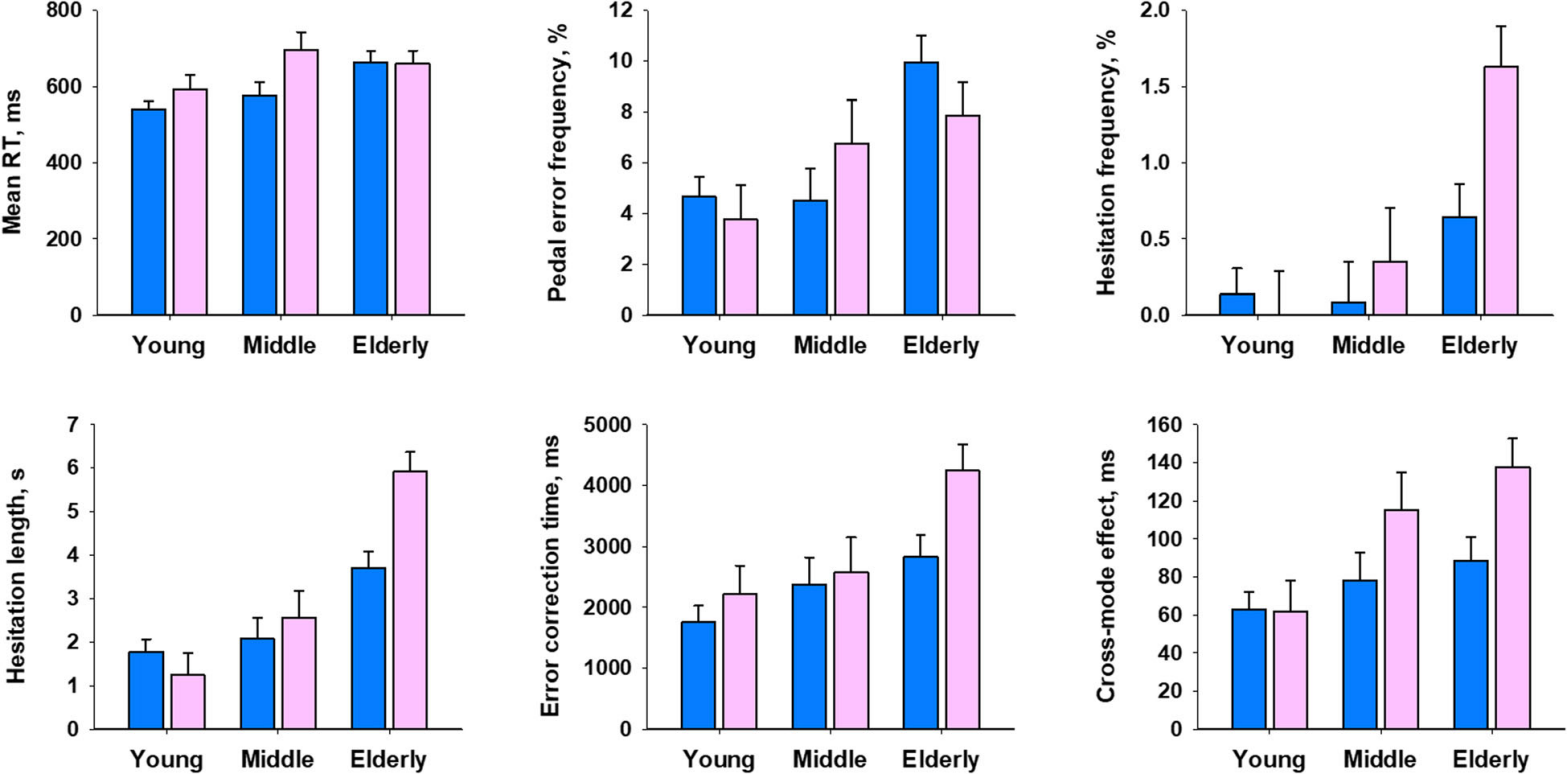
Effects of age and sex on the indicators of PS-PVT. Data are means and the standard errors (error bars). RT = response time

Figure 2 shows the results of comparing the indicators of pedal response characteristics in each sex among the three age groups. Table 3 shows the statistical significance of the effects of age group, sex, and interaction between them. ANOVA revealed significant effects of age group on all PS-PVT indicators. Sex had a significant effect only on a cross-mode effect, showing a greater prolongation in response time (RT) with cross mode in women than men. On average including both men and women, mean RT was 95 ms (17%) longer in the older group than in the young group. More characteristically, however, the older group showed 2.1 times more frequent pedal errors, fell into long hesitations (response freezing > 3 s) 16 times more often, and took 1.8 times longer period for correcting the wrong pedal than the young groups. Although a significant age group-sex interaction was observed for hesitation length, it was due to a significant difference between young and middle-age groups only in women.

**Table 3:** Effects of age and sex on PS-PVT indicators

Indicator	Age		Sex		Age × sex	
	*F*	*P*	*F*	*P*	*F*	*P*
Mean response time	4.97	0.01	4.02	0.05	1.51	0.2
Pedal error frequency	8.65	0.0006	0.05	0.8	1.34	0.2
Hesitation frequency	10.84	0.0001	2.91	0.09	2.79	0.07
Hesitation length	33.52	< 0.0001	3.62	0.06	5.55	0.007
Error correction time	7.94	0.001	3.74	0.05	1.12	0.3
Cross-mode effect	7.40	0.001	5.53	0.02	1.88	0.1

**Fig. 3:**
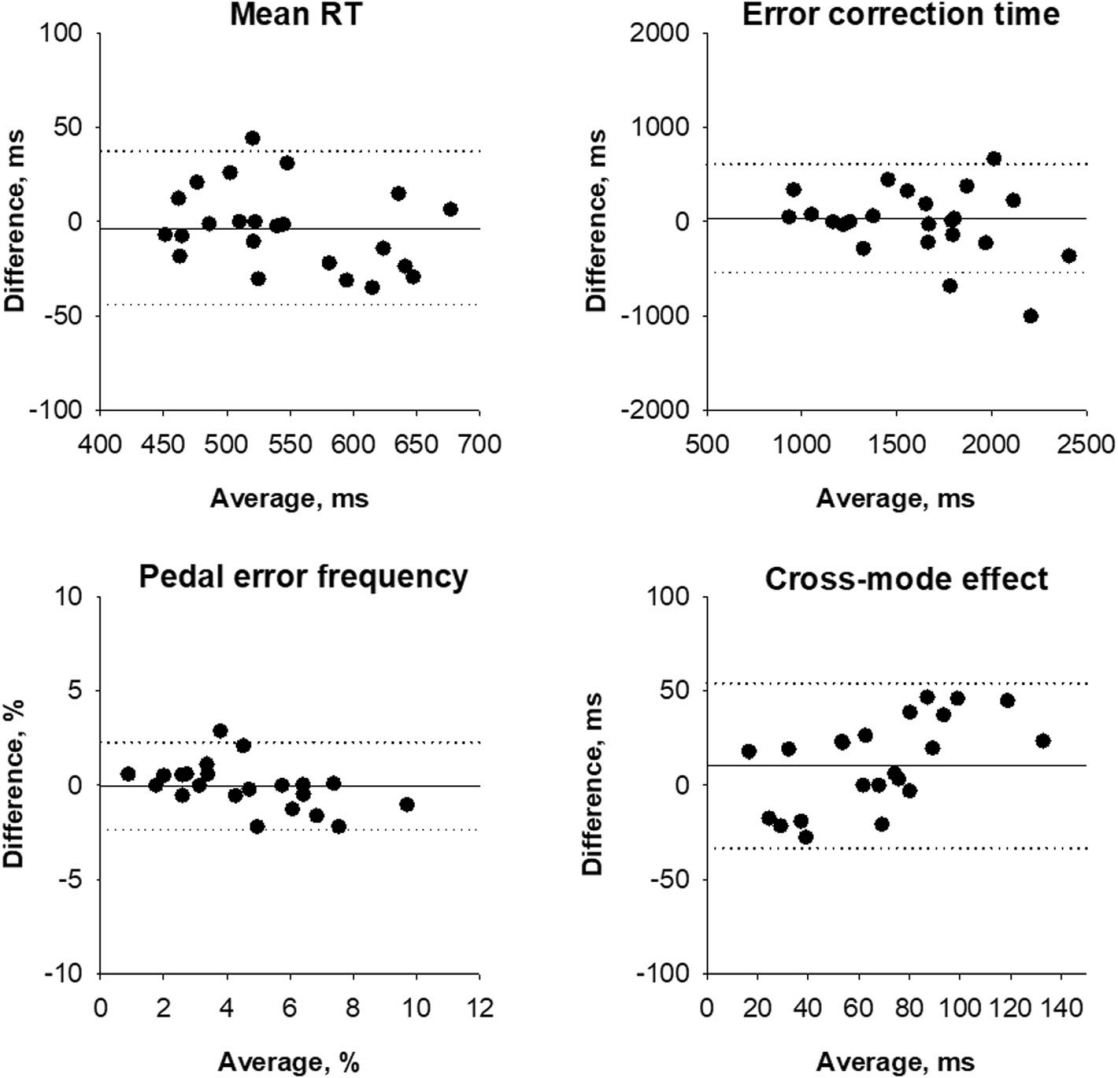
Bland-Altman plots for the repeatability of pedal response characteristic indicators. Solid horizontal lines indicate the mean difference between two trials, and dotted horizontal lines indicate the 95% confidence limits of repeatability

The indicators of pedal response characteristics showed within-individual repeatability to the extent that can identify the age-dependent changes. Figure 3 shows the Bland-Altman plots for the indicators of pedal response characteristics. The repeatability ranges of RT, pedal error frequency, and error correction time were 82 (between − 44 and 37) ms, 4.7 (between − 2.4 and 2.3) %, and 1143 (between − 535 and 608) ms, respectively. These ranges were smaller than the difference between the age groups of these indicators (95 ms, 4.7%, and 1550 ms, respectively).

## Discussion

To examine the hypothesis that pedal operation errors that may be related to unexpected car runaway are more frequently induced in older peoples than in younger peoples, a simple laptop computer system using a foot pedal was developed and the effects of aging on the pedal operation characteristics were examined. The system, named PS-PVT, was able to measure mean RT, pedal error frequency, the frequency and length of hesitation, pedal-error correction time, and cross-mode effect on RT. We applied this system to young, middle-aged, and older subject groups. As a result, mean RT was 95 ms (17%) longer in the older group than in the young group. The older group showed 2.1 times more frequent pedal errors, fell into long hesitations 16 times more often, and took 1.8 times longer period for correcting the wrong pedal than the young groups. We also observed that the indicators of pedal response characteristics showed repeatability to the extent that they can identify age-dependent changes. These findings support the hypothesis that the pedal operation errors that may be related to severe crashes due to unexpected runaway are more frequently induced in older peoples. The PS-PVT may help identify sensorimotor response characteristics associated with crash risk in the elderly population.

Although many studies using driving simulators have demonstrated an age-related decline in driving performance [4–11], those studies also demonstrated a correlation between cognitive performance to understand traffic conditions and driving ability measures. Human cognitive performance is known to decrease with increasing task demand or mental workload [17] and the mental workload during simulated driving increases with the complexity of the scenario [18–20]. This means that measured driving ability and detected degradation by simulators depend on the complexity of the driving scenario. In fact, using driving scenarios with different complexity, Michaels et al. [12] observed that the scenario with medium complexity was best suited to detect differences in driving ability between age groups. These suggest that a simple, scenario-independent task of appropriate complexity may be rather suitable for extracting and quantifying the *absolute* changes in physiological characteristics behind the decline in driving ability with age. PS-PVT is a method that specializes in the ability to apply pedals to visual stimuli. It did not simulate realistic car manipulations or driving situations. Nevertheless, it was able to detect and quantify physiological features that can be associated at least partly with increased crash risk in older drivers.

The PS-PVT was able to identify five distinct pedal response characteristics in the older group; those are (1) longer mean RT, (2) increased pedal error frequency, (3) frequent and prolonged hesitation, (4) extended error correction time, and (5) greater cross-mode effect on RT. Among these, longer mean RT and increased pedal error frequency have been consistently reported in many earlier studies [4–6, 8, 10]. RT and error frequency, however, need to be considered as a trade-off between speed and accuracy [21]. Previous studies have reported that older drivers tend to delay response to improve accuracy, indicating a shift in trade-off point preferring to accuracy [21, 22]. In the present study, however, the older group showed 2.1 times more frequent pedal errors than the younger group, while the RT extension was only 17% compared to the young group. The direction of age-dependent shift in trade-off points may vary depending on task complexity and circumstances such as competitiveness and perceived level of danger.

For the remaining three characteristics, there are at least three points of discussion. First, the hesitation defined as the response freezing > 3 s can be an important feature that may lead to car runaway. It occurred 16 times more frequently in the older group and lasted 4.8 s on average. In a study of the aging effect on RT to traffic lights, Salvia et al. [23] reported a similar phenomenon as no-response (> 2 s). They observed this phenomenon only in the older (> 70 years) group but not in the middle-aged group. It is unclear whether this phenomenon is a physiological characteristic of healthy aging or a pathological symptom of subtle diseases, such as those accompanied by gait freezing [24]. Second, the phenomenon of extended error correction time has not been reported in earlier studies with driving simulators, due probably to the function unique to PS-PVT that requires switching between parallel and cross modes every 2 min. Although this function of PS-PVT seems unrealistic to driving situations, it may be useful for evaluating the ability to avoid serious accidents caused by unexpected runaway, given that the elderly drivers who caused such accidents often reported that they have been confident that the accelerator pedal is a brake pedal. Third, the cross-mode effect on RT is thought to be the Simon effect [25], which is known as a phenomenon that the RT of the reaction in the same direction as the stimuli is shorter than the RT to the reaction in the direction opposite to the stimuli [23]. This phenomenon can also be explained as the well-known effect of extending RT as task complexity increases. Hale et al. [22] reported that this effect increases with age. Our observation of cross-mode effect supports their concept, but also shows that this phenomenon is more pronounced in women than in men.

The present study has both strengths and limitations. The strength is the simplicity, easy-applicability, and time efficiency of the method. The PS-PVT can be performed only in 6 min after a short instruction and practice for < 1 min. It can be used with simple software by Visual Basic, and the hardware requirements are only a commercially available laptop and a foot pedal. Because it provides absolute values of objective physiological characteristics with acceptable reproducibility, the results can be compared across different populations, different societies in traffic environments, and different research conditions. These features are thought to be useful for the widespread use of PS-PVT. On the other hand, the first limitation of this study is that PS-PVT specialized in evaluating pedal application ability among the various functions required for driving. Driving is a complex task achieved by many physiological functions, including sensing, perception, cognition (attention, working memory, and execution), and motor function [26, 27]. Hesitation and extended error correction time may relate to crash risk, but the accidents of vehicle’s crash are resulted not only by that factors but also by many other factors including visual and cognitive function, awareness and fatigue, and spatial control against other cars. A combination with other performance assessments, such as cognitive function tests and scenario-based driving simulators, is necessary to cover a wider range of crash risks. The second limitation of this study is that it does not provide evidence that features extracted in the older group are actually associated with an increased crash risk of elderly drivers. This is the most important theme for future researches. The third limitation is the need for further evaluations of reproducibility. Although we demonstrated that the indicators that showed an inter-group difference had acceptable repeatability in an independent sample, the reproducibility of the inter-group difference itself requires further evaluations. Also, the possibility of improving the indicators through a repetitive training is another important issue for future researches.

## Conclusions

A simple PC system, PS-PVT was developed to quantitatively evaluate the pedal response to visual stimuli. By this system, frequent hesitation and extended error correction time as well as prolonged RT and increased error frequency were identified as the pedal response characteristics in elderly subjects. These may provide insights into a possible mechanism associated with increased crash risk in the elderly population.

## Data Availability

The software of PS-PVT is available from the corresponding author on a reasonable request. Also, the datasets used and/or analyzed during the current study are available from the corresponding author on reasonable request.

## Abbreviations

PS-PVT: Pedal selective psychomotor vigilance test
RT: Response time
